# Enhancing metabolite coverage using dedicated mobile phases for individual polarity modes in HILIC-MS

**DOI:** 10.1007/s00216-025-06189-0

**Published:** 2025-11-19

**Authors:** Alena Langová, Malena Manzi, Jana Brejchová, Ondřej Kuda, Michal Holčapek, Robert Jirásko

**Affiliations:** 1 Department of Analytical Chemistry, Faculty of Chemical Technology, University of Pardubice, Studentská 573, 53210 Pardubice, Czech Republic; 2 Institute of Physiology of the Czech Academy of Sciences, Videnska 1083, 14200 Prague, Czech Republic

**Keywords:** HILIC, Metabolomics, Mass spectrometry, Bioinert chromatography, Metabolite annotation

## Abstract

We have developed a new hydrophilic interaction liquid chromatography-mass spectrometry (HILIC-MS) method with mobile phases optimized for high metabolite coverage in individual polarity modes. This dual mobile phase strategy expands the range of annotated metabolites and improves identification confidence, providing broader and more accurate metabolic profiles. The incorporation of a bioinert chromatographic system further enhances sensitivity. The bridged ethyl hybrid (BEH) amide column yields the best results for metabolomic analysis among the three chromatographic columns in this comparison. The method development involves investigating the effects of mobile phase composition, pH, and a medronic acid additive on the MS response under bioinert chromatographic conditions. The results highlight the important role of alkaline pH for the sensitive detection of polyphosphorylated metabolites, while demonstrating the redundancy of chelating additives in a fully bioinert system. Finally, the optimized method is applied to mouse plasma, pancreas, and liver samples to demonstrate its versatility and reliable performance in complex biological matrices, establishing it as a powerful tool for confident and reproducible metabolomics studies.

**Graphical abstract:**

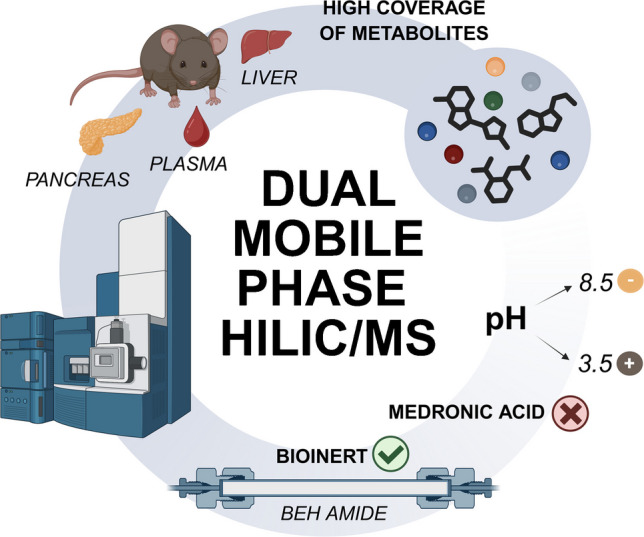

**Supplementary Information:**

The online version contains supplementary material available at 10.1007/s00216-025-06189-0.

## Introduction

A major analytical challenge in MS-based metabolomics is the comprehensive analysis of metabolites with varying polarities, concentration ranges, and diverse physicochemical properties [1, 2]. These complexities are intensified by challenges in the MS ion source, including ionization suppression and in-source fragmentation, particularly for highly polar or unstable compounds like phosphates [3–6]. In metabolomics, ultrahigh-performance liquid chromatography (UHPLC) combined with electrospray (ESI) quadrupole-time-of-flight (QTOF) MS is commonly used due to its high chromatographic resolution, rapid data acquisition, good sensitivity, and precise mass accuracy in MS and MS/MS mode, making it one of the best alternatives for holistic metabolite profiling [7–9].

Reversed-phase liquid chromatography (RP-LC) and hydrophilic interaction liquid chromatography (HILIC) are commonly used for liquid chromatography-mass spectrometry (LC/MS) metabolomic analyses. While RP-LC/MS is primarily applied to less polar metabolites, HILIC-MS remains a versatile technique for the comprehensive analysis of small, polar, and ionizable molecules within biological systems [10]. The core principle of HILIC involves partitioning between an aqueous layer partially immobilized on a hydrophilic stationary phase and a more hydrophobic mobile phase, with electrostatic interactions and hydrogen bonding also contributing to analyte retention depending on the stationary phase properties [11].

The primary advantage of HILIC in metabolomics is its ability to enhance the retention of polar metabolites, enabling the separation and improving the analysis of compounds essential for understanding cellular processes, such as energy metabolism, signaling pathways, and oxidative stress [12]. These metabolites are often crucial for disease diagnosis, biomarker discovery, and the elucidation of disease mechanisms. HILIC is also highly compatible with biological samples rich in water, including plasma, urine, and tissues [13, 14], allowing efficient analysis of polar metabolites directly from these biological matrices without extensive extraction or derivatization [15]. Consequently, HILIC simplifies sample preparation, increases analytical throughput, and broadens the scope of metabolomics and other high-throughput workflows [12, 16].

It is well known that polar compounds containing chromatographically problematic functional groups (e.g., phosphate or sulphate) can interact with metals, especially Fe^3+^ ions at the surface of stainless steel parts of the LC system, leading to poor peak shapes and reduced detection sensitivity [4, 17]. During the chromatographic analysis, the column hardware accounts for over 70% of the surfaces responsible for the interactions with analytes [18]. All components of the LC system, including the column body, tubing, and frits, can contribute to non-specific adsorption of ionic analytes. Several techniques have been developed to reduce this undesirable phenomenon, with conventional methods typically involving a pre-analysis conditioning process. One example is the selective elution of phosphate compounds using titanium dioxide (TiO₂), followed by the derivatization of ionic groups. This multi-step process typically involves the initial activation of TiO₂ particles, the adsorption of phosphate compounds on the activated surface, and subsequent selective elution under basic conditions, commonly using 1–8% ammonium hydroxide [19]. This approach enables the selective capture of phosphorylated metabolites while eliminating substances that could interfere with the analysis [20]. Alternatively, derivatization can be used to enhance the stability of analytes during ionization and potentially increase the signal intensity [15].

Another strategy to minimize metal-analyte interactions is to passivate the entire LC/MS system flow path using phosphoric or citric acid washes [18], either by performing dedicated washing steps or by extending the wash procedure to include the column itself. Recently, several vendors introduced bioinert chromatography systems, in which internal surfaces are fully covered by bioinert materials. Such bioinert configurations offer a more robust and efficient solution than traditional passivation, preventing non-specific interactions with column hardware and improving the analysis of chromatographically challenging analytes. We recently demonstrated the significant benefits of bioinert materials in metabolomics, showing their strong positive impact on sensitivity and peak shape, particularly for CoA and nucleotides [17].

The most common bioinert column types for HILIC separations are the classic silica-, diol-, and amide-based columns. There are also dedicated bioinert columns tailored for specific applications, such as glycans, peptides, or oligonucleotides [21, 22]. Bioinert columns are designed to provide high chemical inertness, minimize adsorption, resist corrosion, and be compatible with a wide range of analytical techniques. Titanium, PEEK, parylene, zirconium, and ceramics, or combinations of these materials, are commonly used in bioinert columns [17, 23].

Given the inherent complexity of metabolomics, obtaining comprehensive coverage of chemically diverse metabolites remains a significant analytical challenge. Specifically, the analysis of highly polar and ionic compounds using HILIC is often hampered by poor peak shape due to undesirable secondary interactions, a problem that modern bioinert hardware aims to solve. However, systematic and comparative evaluations of different bioinert stationary phases in this context remain limited. Furthermore, due to the diverse acid-base properties of metabolites, a single mobile phase pH often represents a suboptimal compromise, necessitating polarity-specific optimization for broad coverage [24]. Finally, careful optimization of the method is crucial to ensure reliable detection of analytes in diluted matrices. This is particularly important in biomedical studies, where limited sample volumes make multiple parallel extractions for multi-omics approaches challenging, highlighting the need for integrated analytical strategies. A suitable solution is two-phase extraction, such as the Folch procedure, which yields both lipid and polar metabolite fractions from the same initial sample volume, allowing their independent use for metabolomic and lipidomic analyses.

This study therefore addresses these challenges by: (i) systematically comparing three distinct bioinert HILIC columns to identify the optimal stationary phase; (ii) developing a dual mobile phase strategy with independently optimized conditions for positive and negative ion modes; and (iii) demonstrating the method’s practical utility for analyzing the polar metabolite fraction from a Folch extract of diverse mouse samples, a workflow that preserves the organic fraction for parallel lipidomic studies.

## Materials and methods

### Chemicals

Acetonitrile (ACN), water, methanol (MeOH), ammonium acetate (AmAc), ammonium formate (AmFo), acetic acid, and formic acid (all LC/MS gradient grade) were purchased from Honeywell (Charlotte, NC, USA). Chloroform (LiChrosolv) was purchased from Merck (Darmstadt, Germany), while medronic acid (Infinity Lab Deactivator Additive) was purchased from Agilent Technologies (Santa Clara, CA, USA). The ammonium solution was obtained from Sigma-Aldrich (St. Louis, MO, USA). Deionized water was prepared using a Milli-Q Reference Water Purification System (Molsheim, France).

### Standards

All standards of metabolites were purchased from Sigma-Aldrich (St. Louis, MO, USA), and standards of lipids were purchased from Avanti Polar Lipids (Alabaster, AL, USA). Stock solutions were prepared by dissolving each metabolite in the appropriate solvent mixture to achieve a final concentration of 1 mg/mL. Solvents were selected based on the solubility of metabolites as follows: water, methanol/acetonitrile (1:1, v/v), acetonitrile/water (2:1; v/v), or chloroform/methanol (1:1; v/v). The standard mixture was prepared by mixing all standards at appropriate concentrations (Table S1), and the final volume was adjusted with acetonitrile/water (1:1; v/v).

### Mouse models for pancreatic ductal adenocarcinoma

Plasma, liver, and pancreatic tissue samples were collected from mice. The pancreatic ductal adenocarcinoma (PDAC) model was established by injecting Panc02 cells (2 × 10^5^) into the pancreas of 7-week-old C57BL/6J male mice obtained from Charles River Laboratories (Sulzfeld, Germany) [25]. The mice were kept at 22 °C on a 12-h light/dark cycle with ad libitum access to food and water. Samples were obtained from control (Dulbecco’s modified Eagle medium (DMEM), *n* = 8) and PDAC mice at early (*n* = 8, day 11) and late (*n* = 7, day 17) stages. All experiments were approved by the Animal Care and Use Committee of the Institute of Physiology (53–2023-P) and were conducted per the Animal Protection Law of the Czech Republic and the European Community Council directives 86/609/EEC. All possible efforts were made to minimize the suffering of the animals. EDTA plasma, liver (distal/proximal to pancreas), and pancreas were collected in a set order by one technician; tissue samples were snap-frozen in liquid nitrogen and stored at −80 °C, as well as plasma samples.

### Tissue cutting and bead-based homogenization

Tissue crushing was performed on a metal block immersed in liquid nitrogen to prevent tissue thawing and enzymatic activity that could affect metabolite concentration levels. The tissue samples were placed in polypropylene Eppendorf tubes, weighed, and snap-frozen in liquid nitrogen before storage at −80 °C. Liver and pancreatic tissues were homogenized in 90% methanol using soft and hard Precellys lysing kits, respectively, with a final concentration of 30 mg/mL. Homogenization was carried out with the Precellys® Evolution Touch tissue homogenizer and Cryolys Evolution cooling system (Bertin Technologies, France), maintaining a constant temperature of 4 °C. The liver was processed at 5600 rpm for two 15-s cycles, with 30-s pauses to prevent overheating. Pancreatic tissue was homogenized at 8000 rpm for three 20-s cycles, also with 30-s pauses. Homogenates were then placed in a water-ice bath, and the metabolite extraction was performed immediately.

### Metabolite two-phase extraction

Polar metabolites were extracted from plasma, liver, and pancreas homogenates using a modified Folch method [26, 27]. Plasma (15 μL) or tissue homogenates (100 μL, equivalent to 3 mg) were mixed with 12 μL of IS mix, 2 mL of chloroform, and methanol (1000 μL for plasma; 900 μL for liver and pancreas) in a 4-mL glass vial, followed by vortexing for 10 s. Tissue samples were then incubated on ice for 30 min. After a 15-min ultrasonic bath, 600 μL of Milli-Q water was added to all samples, and then samples were shaken at 560 rpm (plasma) or 720 rpm (liver and pancreas) for 5 min (IKA KS 130), followed by a centrifugation step performed at 4000 rpm (plasma) or 6000 rpm (liver and pancreas) for 5 min at ambient temperature. The lower organic phase was collected for lipidomic analysis. Subsequently, the secondary extraction was performed by adding 2 mL of CHCl_3_ to the remaining aqueous layer. After shaking and subsequent centrifugation using the same parameters, the second organic phase was collected. The remaining aqueous phase was transferred to a 1.5-mL tube and centrifuged at 12,000 rpm at 4 °C, and the supernatant was transferred to the glass vial and subjected to nitrogen stream evaporation at room temperature. Before analysis, metabolite residues were resuspended in ACN:MeOH (1:1, v/v) and vortexed for 1 min, ensuring thorough homogenization.

For the purpose of demonstrating the method’s applicability in this study, a single pooled sample was prepared for each matrix type. This was achieved by combining equal volumes of the final resuspended polar extracts from all individual samples of a given matrix (i.e., all plasma extracts were pooled, all liver extracts were pooled, and all pancreas extracts were pooled). These three resulting pooled samples were then analyzed by HILIC-MS/MS.

### HILIC-MS analysis

The HILIC-MS analysis was performed using a Bioinert Acquity Premier liquid chromatograph (Waters, Milford, MA, USA) coupled to one of two mass spectrometers.

Initial method development, optimization, and column comparison were conducted on a XEVO G2-XS QTOF mass spectrometer (Waters). The analysis of biological mouse samples was subsequently performed on a timsTOF Ultra mass spectrometer (Bruker Daltonik GmbH, Bremen, Germany) to achieve higher sensitivity.

#### Column comparison and mobile phase optimization

To establish the final optimal method, a multi-step optimization process was performed using the standard metabolite mixture. First, a column comparison was conducted to select the optimal stationary phase. Three distinct bioinert HILIC columns from Waters with identical dimensions (150 × 2.1 mm; 1.7 µm) were evaluated: Acquity Premier BEH HILIC, Atlantis Premier Z-HILIC, and Acquity Premier BEH Amide. This comparison was performed at two pH levels (5.5 and 8.5) in both polarity modes. Second, based on the superior performance of the BEH Amide column, the mobile phase conditions were further optimized. To maximize metabolite coverage, the mobile phase conditions were systematically optimized for each polarity mode separately. The effects of buffer additives were compared by analyzing the standard mixture with mobile phases containing either 15 mM AmAc or 15 mM AmFo at a fixed pH of 5.5.

The influence of pH was investigated using the BEH Amide column. The mobile phase, containing 15 mM of the selected buffer, was adjusted across a pH range of 3.5 to 10 using formic acid or ammonium hydroxide. Measurements were conducted in both polarity modes to assess the effect on retention behavior and MS response for a diverse set of standards. This set included representative metabolites from multiple groups: amino acids (basic, neutral, and acidic), nucleobases, nucleotides, nucleosides, carbohydrates, carnitines, and coenzymes.

#### Final optimized chromatographic conditions

The optimization process described above resulted in the following final chromatographic conditions, which were used for all subsequent analyses. The chromatographic separation was performed on an Acquity Premier BEH Amide column (150 × 2.1 mm, 1.7 µm). The mobile phase A was water containing either 15 mM ammonium formate adjusted to pH 3.5 (for positive polarity mode) or 15 mM ammonium acetate adjusted to pH 9 (for negative polarity mode), and the mobile phase B was acetonitrile (ACN). The gradient program was set as follows: 0 min—95% B; 2 min—85% B; 10 min—60% B; 13 min—50% B; 13.1 min—95% B; 16 min—95% B.

#### Mass spectrometry conditions

The detailed mass spectrometry parameters for the Waters XEVO G2-XS QTOF instrument are provided in Table S2a. Data were acquired in the continuum mode in the m/z range of 50–1200 with the scan time of 0.5 s. Leucine enkephalin was used as the lock mass for real-time mass correction.

A complete list of settings for the Bruker timsTOF Ultra is available in Table S2b. To ensure high mass accuracy on this instrument, a post-run calibration was performed at the end of each analysis by diverting the LC eluent to waste and introducing a sodium formate solution into the ion source via direct infusion. The trapped ion mobility spectrometry function was deactivated to maximize ion transmission and signal intensity. MS/MS experiments were measured using Auto MS/MS mode, and particular parameters are listed in Table S2c.

### Data processing

To reduce background noise in the acquired data, the Waters Compression and Archival tool was used during optimization measurements. The m/z correction was then performed using the LockMass value, and data were converted to centroid mode using the Accurate Mass Measure tool in MassLynx software (Waters). This process reduced file size and minimized the risk of peak misidentification. Peak intensities were evaluated using TargetLynx (Waters) with a predefined mass window tolerance (± 10 mDa).

**Fig. 1:**
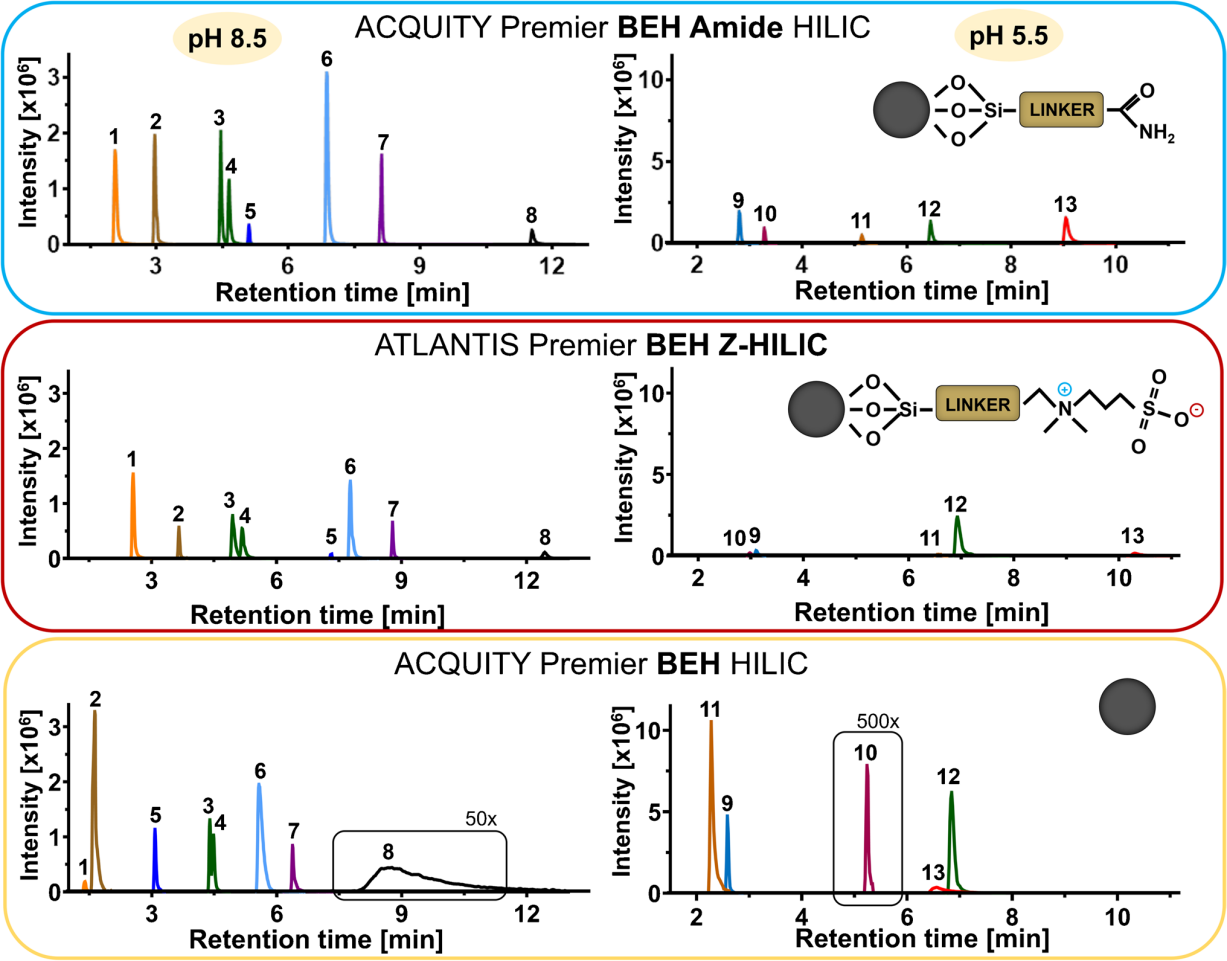
Comparison of three bioinert HILIC columns at two different pH levels (5.5 and 8.5). The chromatograms were reconstructed from full-scan MS data acquired in negative ion mode. The numbered peaks correspond to the following metabolites: (1) uracil, (2) uridine, (3) leucine, (4) isoleucine, (5) taurine, (6) glutamine, (7) acetyl-CoA, (8) GTP, (9) 2-deoxyadenosine, (10) decanoyl-CAR, (11) xanthosine, (12) L-CAR, (13) ADP

**Fig. 2:**
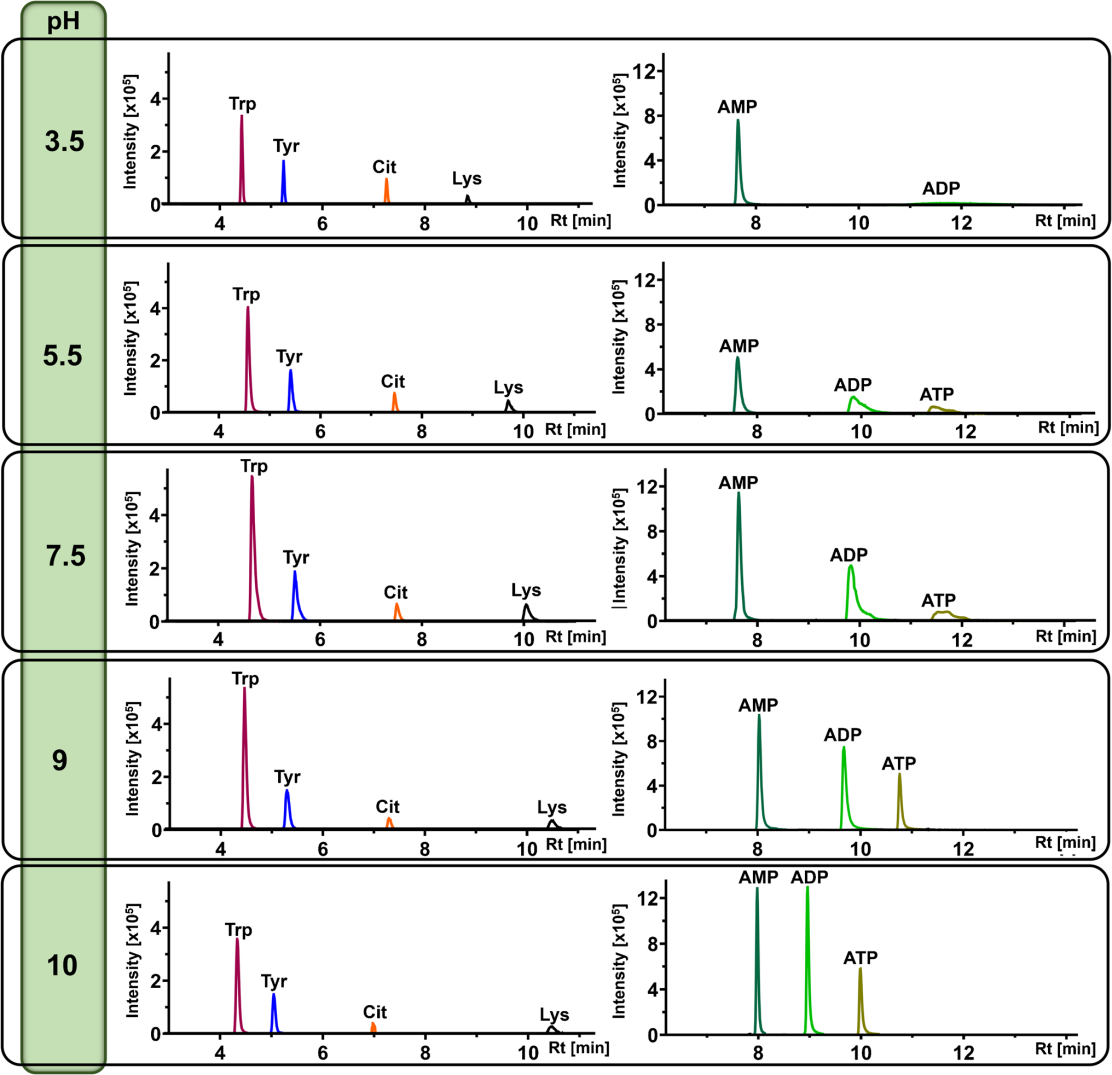
Effect of mobile phase pH (ranging from 3.5 to 10) on the signal of selected metabolites in negative ion mode. The chromatograms were reconstructed from full-scan MS data. Metabolites are abbreviated as follows: Trp, tryptophan; Tyr, tyrosine; Cit, citrulline; Lys, lysine; AMP, adenosine 5′-monophosphate; ADP, adenosine 5′-diphosphate; ATP, adenosine 5′-triphosphate

**Fig. 3:**
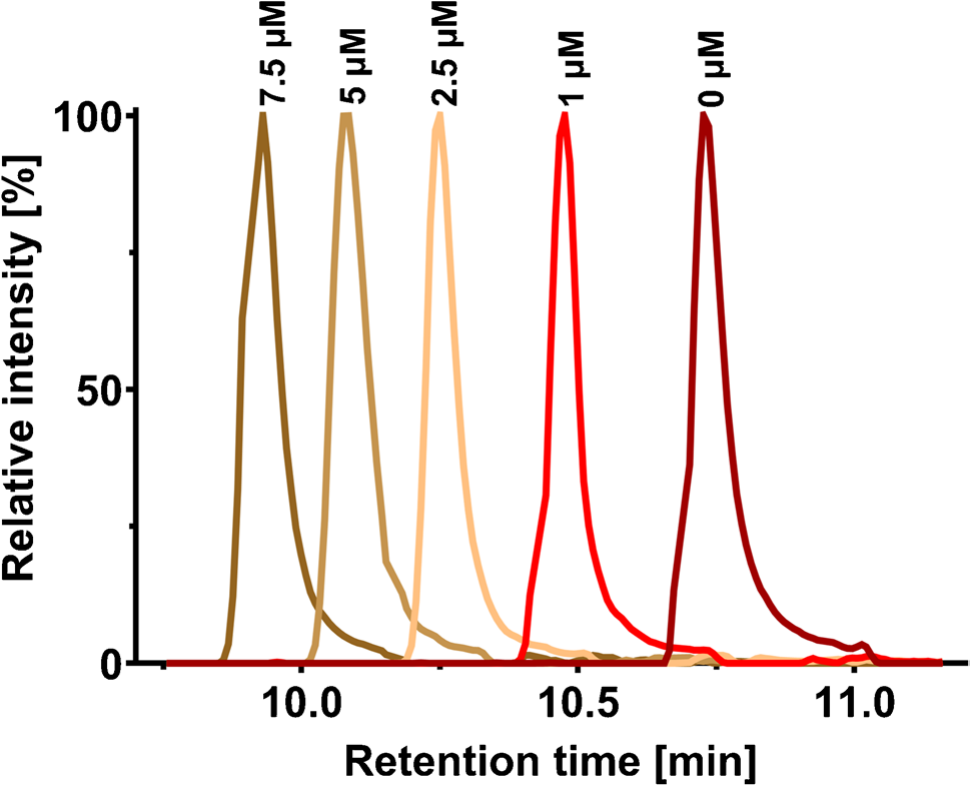
Influence of medronic acid concentration (0–7.5 µM) on the chromatographic peak of uridine 5′-triphosphate in negative ion mode

**Fig. 4:**
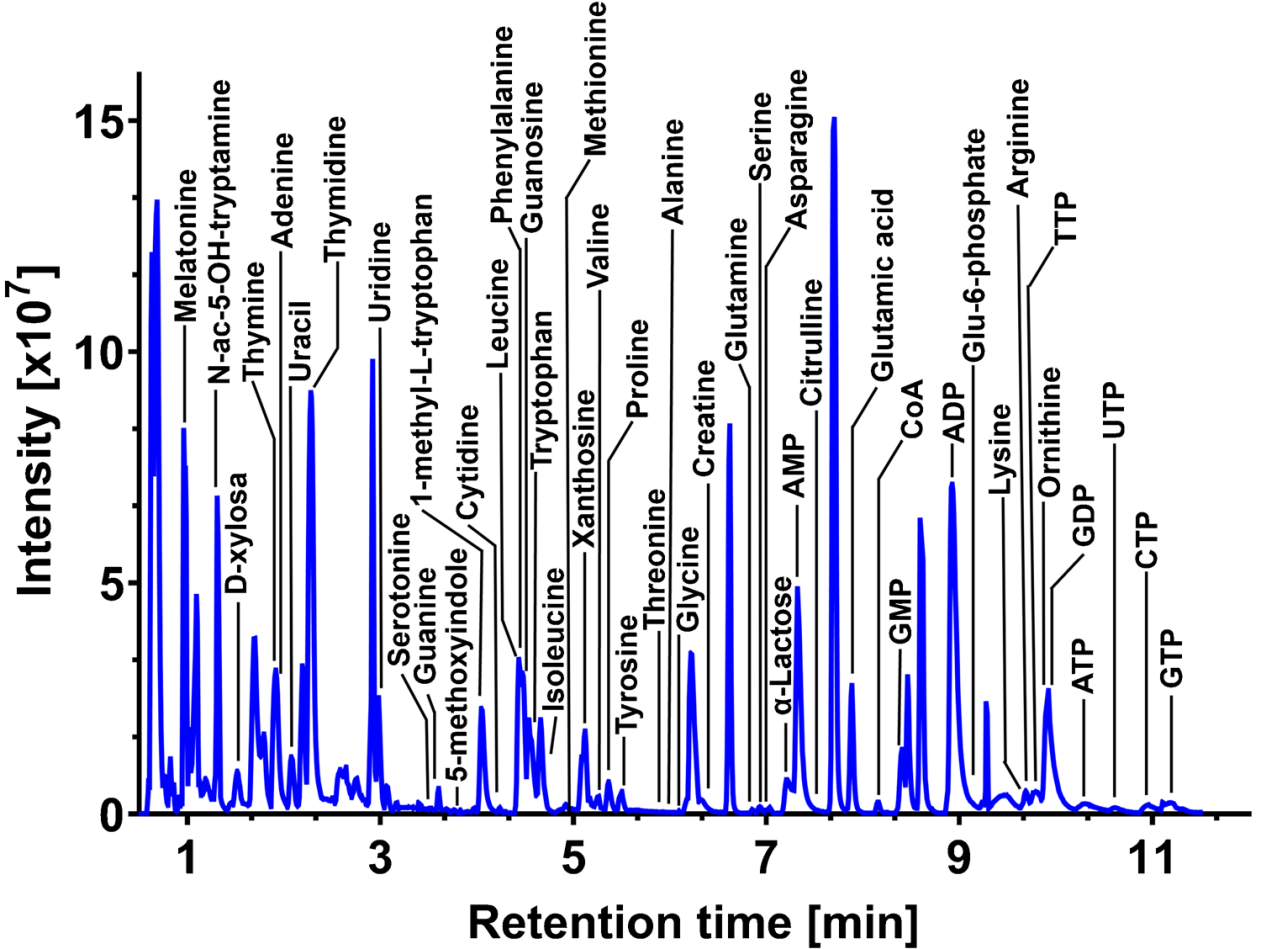
Representative base peak chromatogram (BPC) of a standard metabolite mixture, reconstructed from full-scan MS data under the final optimized HILIC/MS conditions in negative ion mode. Annotated peaks represent a selection of the analyzed metabolites

For the data acquired on the Bruker platform, a separate processing workflow was used. Compass DataAnalysis (Bruker Daltonik GmbH) was employed for post-acquisition calibration using sodium formate and for annotation of real samples. Three types of databases were used to confirm the MS/MS spectral annotations: the Human Metabolome Database (HMDB) [28], MetFrag [29] and an in-house database. A mass accuracy threshold of 5 ppm was used to determine identification precision. The plots in Figs. 1, 2, 3, and 4 were generated using GraphPad Prism (version 10.2.1; GraphPad Software, Boston, MA, USA). No smoothing or other data manipulation was applied. Box plot visualizations (Figures S1 and S2) were created in R (version 4.4.2; R Core Team, Vienna, Austria; www.R-project.org). Heatmaps (Tables S6 and S7) and all other supplementary figures (Figs. S3–S17) were prepared with Microsoft Office 2021 (Microsoft Corp., Redmond, WA, USA). The graphical abstract and schematic diagrams were created with BioRender.com.

## Results and discussion

### Comparison of selected bioinert HILIC columns

The first step in our method development was to compare the performance of three bioinert HILIC columns with distinct stationary phase chemistries: a bare silica (BEH), a zwitterionic (Z-HILIC), and a hybrid amide (BEH amide). All columns were sourced from a single vendor (Waters) to ensure hardware consistency and minimize inter-manufacturer variability, thus allowing for a direct comparison of the stationary phase performance itself. The columns were compared under bioinert conditions at two different pH levels (5.5 and 8.5). In the current study, the slightly acidic pH 5.5 was selected to align with the conditions used in our previous study [17], while the more basic pH 8.5 was chosen to assess column performance under alkaline conditions. The results of the individual measurements are presented in Figs. 1, S1a, b, and S2a, b.

To illustrate the results, representative metabolites from different groups were selected. All measurements were conducted using a standard mixture with component concentrations as listed in Table S1. The chromatographic separation is shown in Fig. 1, which highlights the impact of the stationary phase on metabolite behavior. The BEH Amide column consistently showed the best overall performance, providing a favorable combination of peak shape and sensitivity. The full width at half maximum (FWHM) analysis (boxplots in Figs. S1–S2, Tables S4–S5) confirmed that it yielded the narrowest peak widths for most metabolites, with retention time variation below 0.6% RSD across five injections (Table S3) at both pH levels. This high chromatographic efficiency benefits both resolution and signal-to-noise ratio. The enhanced chromatographic behavior is likely related to the mixed-mode retention mechanism of the amide phase. In addition to hydrophilic partitioning, the amide functionality can act as both a hydrogen bond donor and acceptor, providing a capacity for more specific interactions with diverse analytes compared to BEH silanol or zwitterionic phases.

In contrast, the Z-HILIC column exhibited slightly inferior results in both peak shape and sensitivity for both polarities. In particular, in the case of nucleotides, a relatively well-defined chromatographic peak was still observed at pH 8.5, although with a slightly larger FWHM (Table S4–S5). However, at pH 5.5, the Z-HILIC column displayed significant chromatographic issues, similar to those observed with the BEH column. Although the BEH column provides high sensitivity for nucleobases and similar metabolites, its overall suitability was the weakest among the columns tested for comprehensive coverage in metabolomic analysis.

### Development of a dual-phase HILIC-MS method

Having established the favorable performance of the BEH Amide column for a broad range of metabolites, we proceeded with a systematic optimization of the mobile phase conditions to maximize both chromatographic resolution and ionization efficiency for each polarity mode separately.

#### Selection of buffer additives

In our comparison of the two most common HILIC additives, AmAc and AmFo, we observed distinct effects on challenging compound classes. While AmFo is often preferred for its higher volatility, AmAc can be advantageous for separating certain polar metabolites due to its broader buffering capacity [30].

Our results confirmed this differential behavior. Although no significant difference was observed for many metabolites like amino acids, a clear improvement in peak shape for phosphorylated substances was evident with AmAc in the negative polarity mode (Fig. S3). In contrast, AmFo facilitated better ionization of coenzymes and carnitines in the positive ion mode. Therefore, to maximize performance for each polarity, AmAc was selected for the negative ion mode and AmFo for the positive ion mode.

#### Influence of mobile phase pH

Our systematic evaluation confirmed that mobile phase pH is a critical parameter influencing both retention and ionization, as is well-established for HILIC-MS [6, 31–33]. We observed diverse and often opposing pH dependencies across multiple metabolite classes, clearly illustrating that a single compromise pH would negatively impact the detection of many compounds (Fig. 2).

The results showed a wide variety of pH optima across different metabolite classes (Figs. S4–S14). While many amino acids and nucleosides performed well under neutral or acidic conditions (Figs. S4–S9), there were notable exceptions. For instance, tryptophan required alkaline pH for optimal ionization in negative mode (Fig. S6). Furthermore, some metabolites, such as 2-deoxyadenosine, were efficiently detected only in positive ion mode, with an optimal signal observed at pH 7.5 (Fig. S10). A particularly pronounced and well-established effect was observed for phosphate-containing metabolites. Our data support that polyphosphorylated compounds like ATP benefit from high pH, with an optimal signal at pH 10, whereas monophosphorylated metabolites like AMP or G6P were less sensitive to pH changes (Figs. 2 and S11).

This chemical diversity in pH optima was further illustrated by other compound classes. Coenzymes such as coenzyme A yielded the highest signal in the mid-pH range (pH 5.5–7.5) (Fig. S12), while permanently charged molecules like l-carnitine were detected most sensitively under strongly acidic conditions (pH 3.5) (Fig. S13). The mobile phase pH also profoundly influenced the separation of saccharides, enabling the resolution of lactose anomers at acidic pH, which coalesced into a single peak at alkaline pH due to rapid mutarotation (Fig. S14).

These divergent requirements suggest the potential benefit of a dual-phase strategy. Based on the comprehensive results presented in the heatmaps (Tables S6–S7), we selected pH 9 for negative ionization mode and pH 3.5 for positive ionization mode as the optimal conditions to achieve the broadest possible metabolite coverage.

#### Effect of medronic acid in bioinert LC/MS analysis

Additives such as citrate [34], ethylenediaminetetraacetic acid (EDTA) [35], acetylacetone, or medronic acid (methylenediphosphonic acid) are commonly employed in conventional LC systems to suppress unwanted analyte interactions with active metal surfaces [36, 37]. Their use has been shown to improve peak shape and enhance sensitivity, particularly for phosphate-containing compounds. Modern bioinert hardware is designed to largely prevent these interactions. To experimentally assess the performance of our fully bioinert setup, comprising both the LC system and the column, we systematically examined the effect of medronic acid. Guided by reported optimal concentrations [36, 37], we evaluated the concentration range of 0–7.5 µM in triplicate. As expected, in the fully bioinert system, the addition of medronic acid was found to have no significant effect on the peak shape or intensity of phosphate-containing metabolites (Figs. 3 and S15a, b). FWHM remained consistent across all tested concentrations, with only the retention time varying as the medronic acid concentration increased in the mobile phase (a classic ion-pairing effect). These results provide direct experimental confirmation that the primary benefit of medronic acid stems from its metal-chelating properties. Consequently, our findings demonstrate that the use of such additives is indeed redundant when using a modern, fully bioinert LC configuration.

The systematic optimization process yielded an efficient and robust HILIC-MS method. Figure 4 shows a representative base peak chromatogram of the standard metabolite mixture analyzed under the final optimized conditions for the negative ion mode. The resulting chromatogram displays symmetrical peaks and baseline separation for most analytes, confirming the high performance of the developed protocol.

### Method applicability: metabolite annotation in mouse samples

While the developed method demonstrates excellent performance in a clean mixture of standards, its true utility is determined by its ability to handle the complexity of biological samples. In this context, reliable metabolite characterization remains a challenge in untargeted metabolomics, where misannotation or incorrect identification can lead to misleading interpretations and compromised data reliability [38]. These errors often stem from insufficient reference standards, ambiguous spectral features, instrumental variability, matrix effects, and co-elution of isobaric or structurally related compounds.

To report our findings with a high degree of confidence and clarity, we have adopted the widely accepted tiered system for metabolite annotation. This framework, based on the original Metabolomics Standards Initiative guidelines [39, 40] classifies identifications based on the strength of the supporting evidence. *Level 1* represents the highest confidence, requiring a direct match of chromatographic retention time and mass spectra (MS and MS/MS) with an authentic chemical standard. *Level 2* is assigned to putative annotations based on MS/MS spectral similarity to public [28, 29] or in-house databases, combined with accurate mass. For homologous series such as fatty acids and acylcarnitines, this level of annotation is supported by their characteristic and predictable chromatographic behavior [41], where retention time systematically correlates with acyl chain length, as illustrated in Fig. S16. *Level 3* relies on characteristic physicochemical properties, such as accurate mass and elemental composition, to suggest a compound class [41].

In this study, the majority of reported metabolites were assigned as *Level 2* annotations, supported by high-resolution MS data and MS/MS fragmentation spectra (mass accuracy < 3 ppm) that matched library data and reflected class-specific structural features (Tables S8–S9). A subset of these were further confirmed as *Level 1* identifications by comparison with authentic standards measured under identical conditions.

To assess its practical applicability, the optimized dual mobile phase workflow was applied to the polar metabolite fraction from Folch extract of mouse plasma, liver, and pancreas. Across all samples, a total of 203 metabolites were confidently annotated (Tables S8–S9). This total number comprises 110 metabolites uniquely annotated in negative ion mode and 62 in positive ion mode, with an additional 31 metabolites detected and confirmed in both modes (Fig. S17). Specifically, 160 metabolites were detected in plasma, 145 in liver, and 151 in pancreas, highlighting the method’s ability to cover a wide range of metabolites in various tissue types. It is important to place these numbers in the context of the study’s specific scope. While they may appear modest compared to some comprehensive human studies, they are influenced by several factors. Firstly, the metabolome of laboratory mice on a standardized diet is inherently less complex than that of a diverse human population, particularly concerning the vast array of exogenous compounds. Secondly, our workflow was specifically designed to analyze the polar metabolite fraction from the Folch extraction, intentionally excluding the nonpolar lipid fraction, which contains hundreds of additional molecular species.

Nevertheless, the coverage achieved by our HILIC-MS method is competitive with current state-of-the-art approaches. For context, many untargeted metabolomic studies report a high number of “molecular features” rather than confidently annotated metabolites. For example, Saarinen et al. identified over 1500 molecular features in mouse plasma, but the subsequent discussion of identified compounds was limited to a small subset of metabolites, with the majority of features remaining unannotated [42]. Similarly, other high-quality studies on mouse serum using modern instrumentation have reported the confident identification of approximately 40 metabolites from a larger pool of features [43]. Even comprehensive studies analyzing multiple mouse samples (plasma, liver, and muscle) with a combination of three analytical platforms (GC/MS and two separate LC/MS methods) have reported the annotation of around 180 polar metabolites [44].

The annotation of 203 metabolites in our study, including a broad range of compound classes such as amino acids, nucleosides, nucleotides, coenzymes, carnitines, organic acids, and carbohydrates (a complete list is provided in Tables S8 and S9), demonstrates the effectiveness of our optimized dual mobile phase strategy.

## Conclusions

This work demonstrates that tailoring mobile phases for individual polarity modes in HILIC-MS broadens coverage for chemically diverse metabolite classes. The selection of buffer and pH is essential for improving retention and detection of metabolites. We achieved marked enhancements in the chromatographic performance based on the independent optimization of the buffer composition using ammonium formate at pH 3.5 for the positive ion mode and ammonium acetate at pH 9 for the negative ion mode and the selection of additives. Our results confirm that optimized HILIC-MS conditions allow for confident annotation of phosphate-containing metabolites. Compounds with multiple free phosphate groups (e.g., ATP) exhibit the most significant improvement of peak shapes and intensity under alkaline pH conditions. Mono-phosphorylated metabolites, such as AMP or G6P, are less pH-sensitive, yet also perform well under moderately basic conditions. Among the tested stationary phases, the BEH Amide column consistently demonstrated superior peak shape, sensitivity, and reproducibility. Furthermore, the use of medronic acid as an ion-pairing additive in a fully bioinert system did not yield additional benefit, highlighting the effectiveness of modern bioinert configurations in minimizing analyte-surface interactions. The application of this dual mobile phase workflow to the polar phase of Folch extracts from mouse plasma, liver, and pancreas enabled confident annotation of 203 metabolites (160 in plasma, 145 in liver, and 151 in pancreas), with a remarkable 54 of these confirmed to *Level 1* specificity using authentic chemical standards. This was based on high-resolution MS and MS/MS fragmentation data (mass accuracy < 3 ppm). The reported metabolite coverage should be viewed within the specific scope of this study, targeting the polar fraction from laboratory mice whose metabolome is inherently less complex than that of humans. Nevertheless, the reliable and comprehensive profiling achieved in these challenging matrices demonstrates the promising potential of the polarity-specific mobile phase design for untargeted HILIC-MS analysis.

## Supplementary Information

Below is the link to the electronic supplementary material.

Supplementary Material 1 Supplementary Figures S1-S17, providing comparative chromatograms, boxplots of column performance, data plots from optimization experiments, correlation analysis of homologous series, and a Venn diagram of metabolite distribution. (DOCX 2.14 MB)

Supplementary Material 2 Supplementary Tables S1-S9, providing the list of standards, instrument parameters, detailed comparison data, heatmaps, and lists of annotated metabolites. (XLSX 130 KB)

## Data Availability

The authors declare that the data supporting the findings of this study is available within the paper and its Supplementary Information files. Raw data files are available from the corresponding author upon reasonable request.

## References

[1] McCullagh J, Probert F. New analytical methods focusing on polar metabolite analysis in mass spectrometry and NMR-based metabolomics. Curr Opin Chem Biol. 2024. 10.1016/j.cbpa.2024.102466. 38772215 10.1016/j.cbpa.2024.102466

[2] Rakusanova S, Cajka T. Tips and tricks for LC–MS-based metabolomics and lipidomics analysis. TrAC Trends Anal Chem. 2024. 10.1016/j.trac.2024.117940.

[3] Mahmud I, Wei B, Veillon L, Tan L, Martinez S, Tran B, Raskind A, de Jong F, Liu Y, Ding J, Xiong Y, Chan WK, Akbani R, Weinstein JN, Beecher C, Lorenzi PL. Ion suppression correction and normalization for non-targeted metabolomics. Nat Commun. 2025. 10.1038/s41467-025-56646-8. 39905052 10.1038/s41467-025-56646-8 PMC11794426

[4] Spalding JL, Naser FJ, Mahieu NG, Johnson SL, Patti GJ. Trace phosphate improves ZIC-pHILIC peak shape, sensitivity, and coverage for untargeted metabolomics. J Proteome Res. 2018;17:3537–46. 10.1021/acs.jproteome.8b00487. 30160483 10.1021/acs.jproteome.8b00487 PMC6427830

[5] Alseekh S, Aharoni A, Brotman Y, Contrepois K, D’Auria J, Ewald JC, Ewald J, Fraser PD, Giavalisco P, Hall RD, Heinemann M, Link H, Luo J, Neumann S, Nielsen J, Perez de Souza L, Saito K, Sauer U, Schroeder FC, Schuster S, Siuzdak G, Skirycz A, Sumner LW, Snyder MP, Tang H, Tohge T, Wang Y, Wen W, Wu S, Xu G, Zamboni N, Fernie AR. Mass spectrometry-based metabolomics: a guide for annotation, quantification and best reporting practices. Nat Methods. 2021;18:747–56. 34239102 10.1038/s41592-021-01197-1 PMC8592384

[6] Brookhart A, Arora M, McCullagh M, Wilson ID, Plumb RS, Vissers JP, Tanna N. Understanding mobile phase buffer composition and chemical structure effects on electrospray ionization mass spectrometry response. J Chromatogr A. 2023. 10.1016/j.chroma.2023.463966. 37054638 10.1016/j.chroma.2023.463966

[7] Zhou B, Xiao JF, Tuli L, Ressom HW. LC-MS-based metabolomics. Mol Biosyst. 2012;8:470–81. 22041788 10.1039/c1mb05350g PMC3699692

[8] Johnson CH, Ivanisevic J, Siuzdak G. Metabolomics: beyond biomarkers and towards mechanisms. Nat Rev Mol Cell Biol. 2016;17:451–9. 10.1038/nrm.2016.25. 26979502 10.1038/nrm.2016.25 PMC5729912

[9] Schuhmacher R, Krska R, Weckwerth W, Goodacre R. Metabolomics and metabolite profiling. Anal Bioanal Chem. 2013;405:5003–4. 23591644 10.1007/s00216-013-6939-5

[10] Wernisch S, Pennathur S. Evaluation of coverage, retention patterns, and selectivity of seven liquid chromatographic methods for metabolomics. Anal Bioanal Chem. 2016;408:6079–91. 10.1007/s00216-016-9716-4. 27370688 10.1007/s00216-016-9716-4 PMC4983217

[11] Nováková L, Havlíková L, Vlčková H. Hydrophilic interaction chromatography of polar and ionizable compounds by UHPLC. TrAC - Trends in Analytical Chemistry. 2014;63:55–64.

[12] Sheng Q, Liu M, Lan M, Qing G. Hydrophilic interaction liquid chromatography promotes the development of bio-separation and bio-analytical chemistry. TrAC Trends Anal Chem. 2023. 10.1016/j.trac.2023.117148.

[13] Buszewski B, Noga S. Hydrophilic interaction liquid chromatography (HILIC)-a powerful separation technique. Anal Bioanal Chem. 2012;402:231–47. 21879300 10.1007/s00216-011-5308-5 PMC3249561

[14] Cubbon S, Antonio C, Wilson J, Thomas-Oates J. Metabolomic applications of HILIC-LC-MS. Mass Spectrom Rev. 2010;29:671–84. 10.1002/mas.20252. 19557839 10.1002/mas.20252

[15] Zhao S, Li L. Chemical derivatization in LC-MS-based metabolomics study. TrAC Trends Anal Chem. 2020. 10.1016/j.trac.2020.115988.

[16] Assress HA, Hameed A, Pack LM, Ferruzzi MG, Lan RS. Evaluation of ion source parameters and liquid chromatography methods for plasma untargeted metabolomics using orbitrap mass spectrometer. J Chromatogr B Analyt Technol Biomed Life Sci. 2025. 10.1016/j.jchromb.2025.124564. 40209549 10.1016/j.jchromb.2025.124564

[17] Peterka O, Langová A, Jirásko R, Holčapek M. Bioinert UHPLC system improves sensitivity and peak shapes for ionic metabolites. J Chromatogr A. 2025. 10.1016/j.chroma.2024.465588. 39662336 10.1016/j.chroma.2024.465588

[18] Gilar M, DeLano M, Gritti F. Mitigation of analyte loss on metal surfaces in liquid chromatography. J Chromatogr A. 2021. 10.1016/j.chroma.2021.462247. 34087520 10.1016/j.chroma.2021.462247

[19] Serafimov K, Lämmerhofer M. Comprehensive coverage of glycolysis and pentose phosphate metabolic pathways by isomer-selective accurate targeted hydrophilic interaction liquid chromatography-tandem mass spectrometry assay. Anal Chem. 2024;96:17271–9. 10.1021/acs.analchem.4c03490. 39425639 10.1021/acs.analchem.4c03490

[20] Garcia-Contreras R, Sugimoto M, Umemura N, Kaneko M, Hatakeyama Y, Soga T, Tomita M, Scougall-Vilchis RJ, Contreras-Bulnes R, Nakajima H, Sakagami H. Alteration of metabolomic profiles by titanium dioxide nanoparticles in human gingivitis model. Biomaterials. 2015;57:33–40. 10.1016/j.biomaterials.2015.03.059. 25913073 10.1016/j.biomaterials.2015.03.059

[21] Rojahn AM, Esser D. HILIC analysis of oligonucleotides using bioinert columns. LCGC Europe 2023;36(6):238–239.

[22] Lardeux H, D’Atri V, Guillarme D. Recent advances and current challenges in hydrophilic interaction chromatography for the analysis of therapeutic oligonucleotides. TrAC -Trends Anal Chem. 2024;176. 10.1016/j.trac.2024.117758.

[23] Murisier A, Fekete S, Guillarme D, D’Atri V. The importance of being metal-free: the critical choice of column hardware for size exclusion chromatography coupled to high resolution mass spectrometry. Anal Chim Acta. 2021;1183:338987. 10.1016/j.aca.2021.338987. 10.1016/j.aca.2021.338987 34627511

[24] Gilar M, Berthelette KD, Walter TH. Contribution of ionic interactions to stationary phase selectivity in hydrophilic interaction chromatography. J Sep Sci. 2022;45:3264–75. 10.1002/jssc.202200165. 35347885 10.1002/jssc.202200165 PMC9545918

[25] Partecke LI, Sendler M, Kaeding A, Weiss FU, Mayerle J, Dummer A, Nguyen TD, Albers N, Speerforck S, Lerch MM, Heidecke CD, von Bernstorff W, Stier A. A syngeneic orthotopic murine model of pancreatic adenocarcinoma in the C57/BL6 mouse using the Panc02 and 6606PDA cell lines. Eur Surg Res. 2011;47:98–107. 10.1159/000329413. 21720167 10.1159/000329413

[26] Idkowiak J, Jirásko R, Kolářová D, Bártl J, Hájek T, Antonelli M, Vaňková Z, Wolrab D, Hrstka R, Študentová H, Melichar B, Pešková K, Holčapek M. Robust and high-throughput lipidomic quantitation of human blood samples using flow injection analysis with tandem mass spectrometry for clinical use. Anal Bioanal Chem. 2023;415:935–51. 10.1007/s00216-022-04490-w. 36598539 10.1007/s00216-022-04490-w

[27] Wolrab D, Chocholoušková M, Jirásko R, Peterka O, Holčapek M. Validation of lipidomic analysis of human plasma and serum by supercritical fluid chromatography–mass spectrometry and hydrophilic interaction liquid chromatography–mass spectrometry. Anal Bioanal Chem. 2020;412:2375–88. 10.1007/s00216-020-02473-3. 32078000 10.1007/s00216-020-02473-3

[28] Wishart DS, Guo AC, Oler E, Wang F, Anjum A, Peters H, Dizon R, Sayeeda Z, Tian S, Lee BL, Berjanskii M, Mah R, Yamamoto M, Jovel J, Torres-Calzada C, Hiebert-Giesbrecht M, Lui VW, Varshavi D, Varshavi D, Allen D, Arndt D, Khetarpal N, Sivakumaran A, Harford K, Sanford S, Yee K, Cao X, Budinski Z, Liigand J, Zhang L, Zheng J, Mandal R, Karu N, Dambrova M, Schiöth HB, Greiner R, Gautam V. HMDB 5.0: the human metabolome database for 2022. Nucleic Acids Res. 2022;50:D622–31. 10.1093/nar/gkab1062. 34986597 10.1093/nar/gkab1062 PMC8728138

[29] Ruttkies C, Schymanski EL, Wolf S, Hollender J, Neumann S. Metfrag relaunched: incorporating strategies beyond in silico fragmentation. J Cheminform. 2016. 10.1186/s13321-016-0115-9. 26834843 10.1186/s13321-016-0115-9 PMC4732001

[30] Garcıa MC, Hogenboom AC, Zappey H, Irth H. Effect of the mobile phase composition on the separation and detection of intact proteins by reversed-phase liquid chromatography-electrospray mass spectrometry. J Chromatogr A. 2002. 10.1016/S0021-9673(02)00345-X 10.1016/s0021-9673(02)00345-x 12113342

[31] Zhang J, Dai J, Wang S, Rui K, Dai W, Kang X, Ji J, Wang W. Evaluation of metabolites and biological activities of Areca nut (*Areca catechu* L.) under different pH: untargeted mass spectrometry-based metabolomics approach. J Food Biochem. 2025. 10.1155/jfbc/9920574.

[32] Chan CCY, Gregson DB, Wildman SD, Bihan DG, Groves RA, Aburashed R, Rydzak T, Pittman K, Van Bavel N, Lewis IA. Metabolomics strategy for diagnosing urinary tract infections. Nat Commun. 2025. 10.1038/s41467-025-57765-y. 40102424 10.1038/s41467-025-57765-y PMC11920235

[33] Zhao Y, Ma C, Cai R, Xin L, Li Y, Ke L, Ye W, Ouyang T, Liang J, Wu R, Lin Y. NMR and MS reveal characteristic metabolome atlas and optimize esophageal squamous cell carcinoma early detection. Nat Commun. 2024. 10.1038/s41467-024-46837-0. 38504100 10.1038/s41467-024-46837-0 PMC10951220

[34] McCalley DV. Influence of metals in the column or instrument on performance in hydrophilic interaction liquid chromatography. J Chromatogr A. 2022. 10.1016/j.chroma.2021.462751. 34995861 10.1016/j.chroma.2021.462751

[35] Wang G, Tomasella FP. Ion-pairing HPLC methods to determine EDTA and DTPA in small molecule and biological pharmaceutical formulations. J Pharm Anal. 2016;6:150–6. 10.1016/j.jpha.2016.01.002. 29403975 10.1016/j.jpha.2016.01.002 PMC5762494

[36] Birdsall RE, Kellett J, Yu YQ, Chen W. Application of mobile phase additives to reduce metal-ion mediated adsorption of non-phosphorylated peptides in RPLC/MS-based assays. J Chromatogr B Analyt Technol Biomed Life Sci. 2019. 10.1016/j.jchromb.2019.121773. 31470201 10.1016/j.jchromb.2019.121773

[37] Hsiao JJ, Potter OG, Chu TW, Yin H. Improved LC/MS methods for the analysis of metal-sensitive analytes using medronic acid as a mobile phase additive. Anal Chem. 2018;90:9457–64. 10.1021/acs.analchem.8b02100. 29976062 10.1021/acs.analchem.8b02100

[38] Xu YF, Lu W, Rabinowitz JD. Avoiding misannotation of in-source fragmentation products as cellular metabolites in liquid chromatography-mass spectrometry-based metabolomics. Anal Chem. 2015;87:2273–81. 10.1021/ac504118y. 25591916 10.1021/ac504118y PMC4354698

[39] Sumner LW, Amberg A, Barrett D, Beale MH, Beger R, Daykin CA, Fan TWM, Fiehn O, Goodacre R, Griffin JL, Hankemeier T, Hardy N, Harnly J, Higashi R, Kopka J, Lane AN, Lindon JC, Marriott P, Nicholls AW, Reily MD, Thaden JJ, Viant MR. Proposed minimum reporting standards for chemical analysis: Chemical Analysis Working Group (CAWG) Metabolomics Standards Initiative (MSI). Metabolomics. 2007;3:211–21. 10.1007/s11306-007-0082-2. 24039616 10.1007/s11306-007-0082-2 PMC3772505

[40] Schymanski EL, Jeon J, Gulde R, Fenner K, Ruff M, Singer HP, Hollender J. Identifying small molecules via high resolution mass spectrometry: communicating confidence. Environ Sci Technol. 2014;48:2097–8. 24476540 10.1021/es5002105

[41] Domingo-Almenara X, Montenegro-Burke JR, Benton HP, Siuzdak G. Annotation: a computational solution for streamlining metabolomics analysis. Anal Chem. 2018;90:480–9. 29039932 10.1021/acs.analchem.7b03929 PMC5750104

[42] Saarinen MT, Kärkkäinen O, Hanhineva K, Tiihonen K, Hibberd A, Mäkelä KA, Raza GS, Herzig K-H, Anglenius H. Metabolomics analysis of plasma and adipose tissue samples from mice orally administered with polydextrose and correlations with cecal microbiota. Sci Rep. 2020;10:21577. 10.1038/s41598-020-78484-y. 33299048 10.1038/s41598-020-78484-y PMC7726573

[43] Goon DE, Ab-Rahim S, Mohd Sakri AH, Mazlan M, Tan JK, Abdul Aziz M, Mohd Noor N, Ibrahim E, Sheikh Abdul Kadir SH. Untargeted serum metabolites profiling in high-fat diet mice supplemented with enhanced palm tocotrienol-rich fraction using UHPLC-MS. Sci Rep. 2021;11:21001. 10.1038/s41598-021-00454-9. 34697380 10.1038/s41598-021-00454-9 PMC8546078

[44] Giesbertz P, Padberg I, Rein D, Ecker J, Höfle AS, Spanier B, Daniel H. Metabolite profiling in plasma and tissues of ob/ob and db/db mice identifies novel markers of obesity and type 2 diabetes. Diabetologia. 2015;58:2133–43. 10.1007/s00125-015-3656-y. 10.1007/s00125-015-3656-y 26058503

