# Supplementary material for: Enhancing metabolite coverage using dedicated mobile phases for individual polarity modes in HILIC-MS
Source: Anal Bioanal Chem. 2025 Nov 19;418(2):747–57. doi: 10.1007/s00216-025-06189-0 (PMC12783170; doi:10.1007/s00216-025-06189-0)

**Electronic Supplementary Material 1 (ESM 1)**

**Enhancing Metabolite Coverage using Dedicated Mobile Phases for Individual Polarity Modes in HILIC/MS**

Langová Alena^1^, Malena Manzi^1^, Jana Brejchova^2^, Ondřej Kuda^2^, Michal Holčapek^1^, Robert Jirásko^1^*

*^1^ University of Pardubice, Faculty of Chemical Technology, Department of Analytical Chemistry, Studentská 573, 53210 Pardubice, Czech Republic*

*^2^ Institute of Physiology of the Czech Academy of Sciences, Videnska 1083, Prague, 14200, Czech Republic*

*Corresponding author: Robert Jirásko, Tel.: +420 466 037 089; Fax: +420 466 037 068; Email: [robert.jirasko@upce.cz](mailto:robert.jirasko@upce.cz)

**Figure S1** Comparison of three bioinert column performance in negative polarity mode using boxplot analysis **(A)** pH 5.5 **(B)** pH 8.5

**(A)**


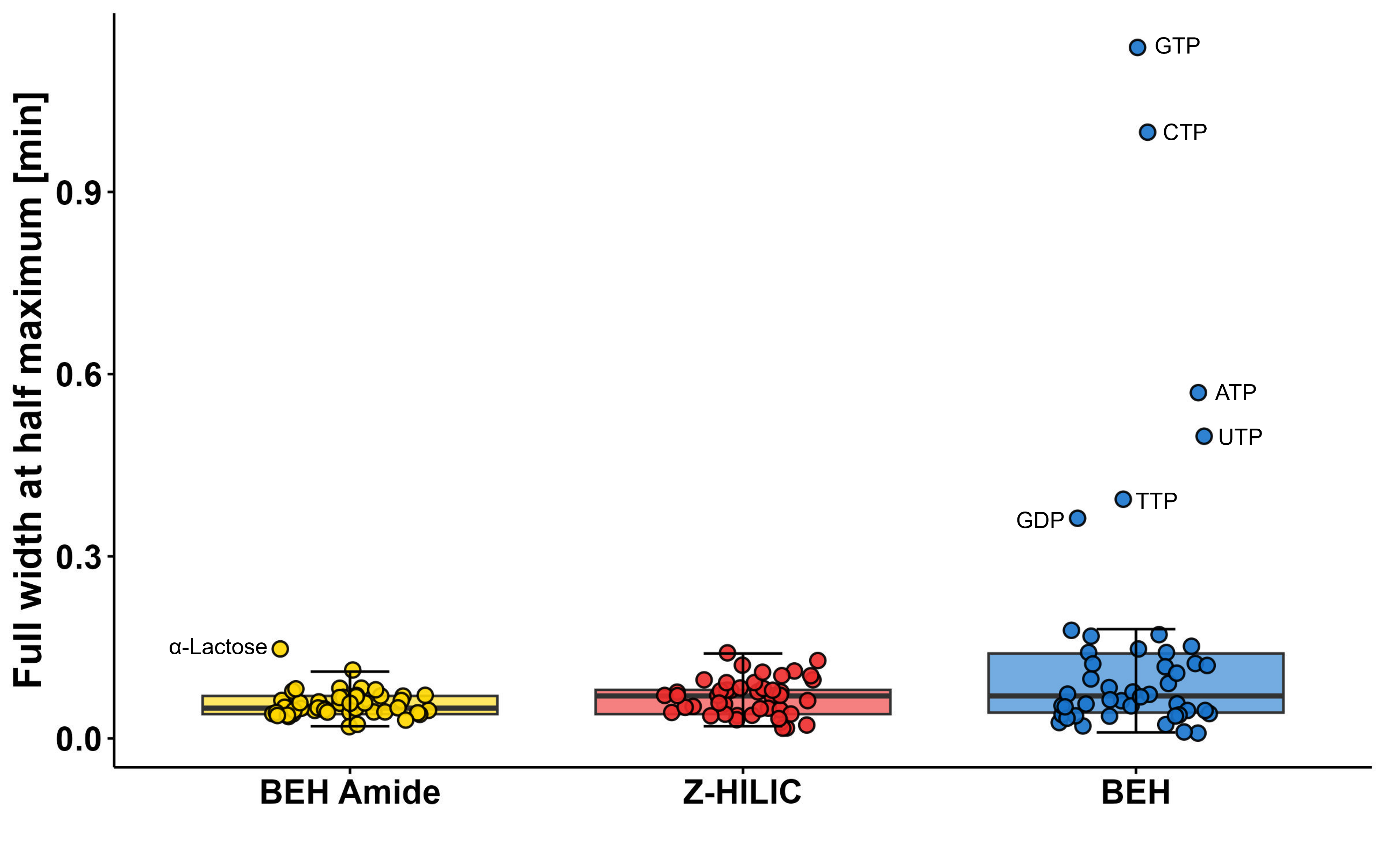

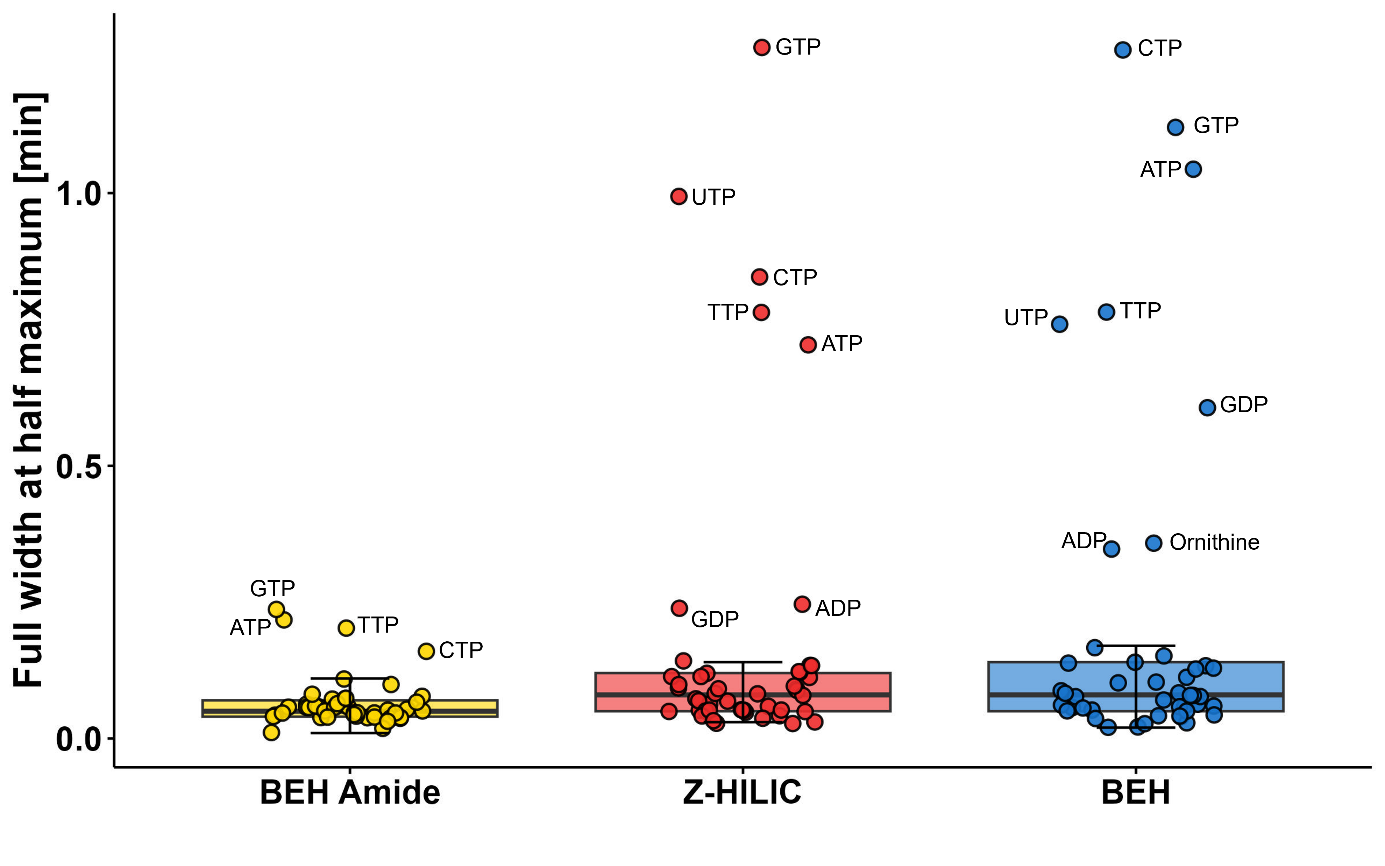


**(B)**

**Figure S2** Comparison of three bioinert column performance in positive polarity mode using boxplot analysis **(A)** pH 5.5 **(B)** pH 8.5

**(A)**

**
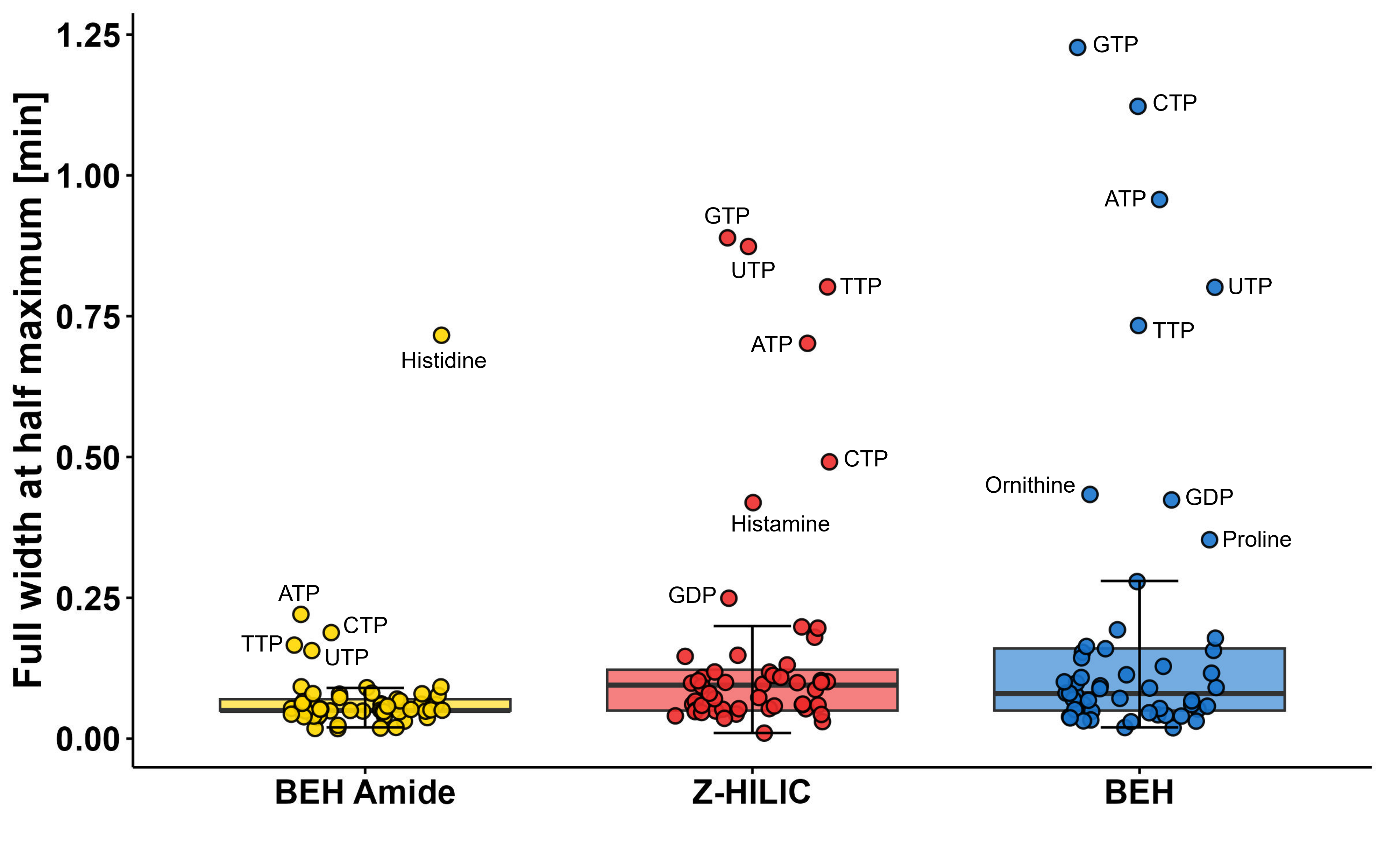
**

**(B)**

**
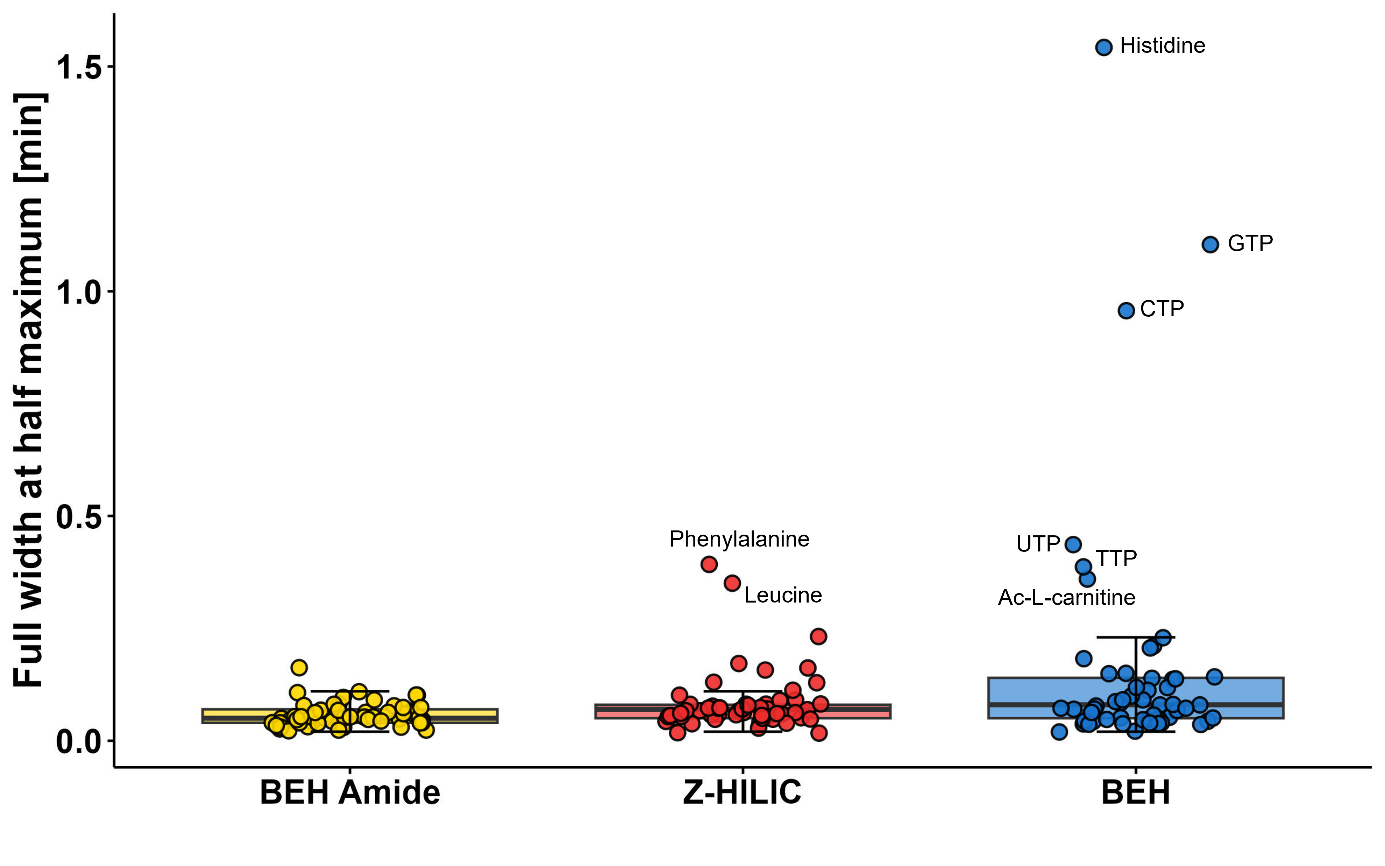
**

**Figure S3** Effect of mobile phase additives, **c**omparison of 15 mM AmAc with 15 mM AmFo for ionization of metabolites in negative polarity mode


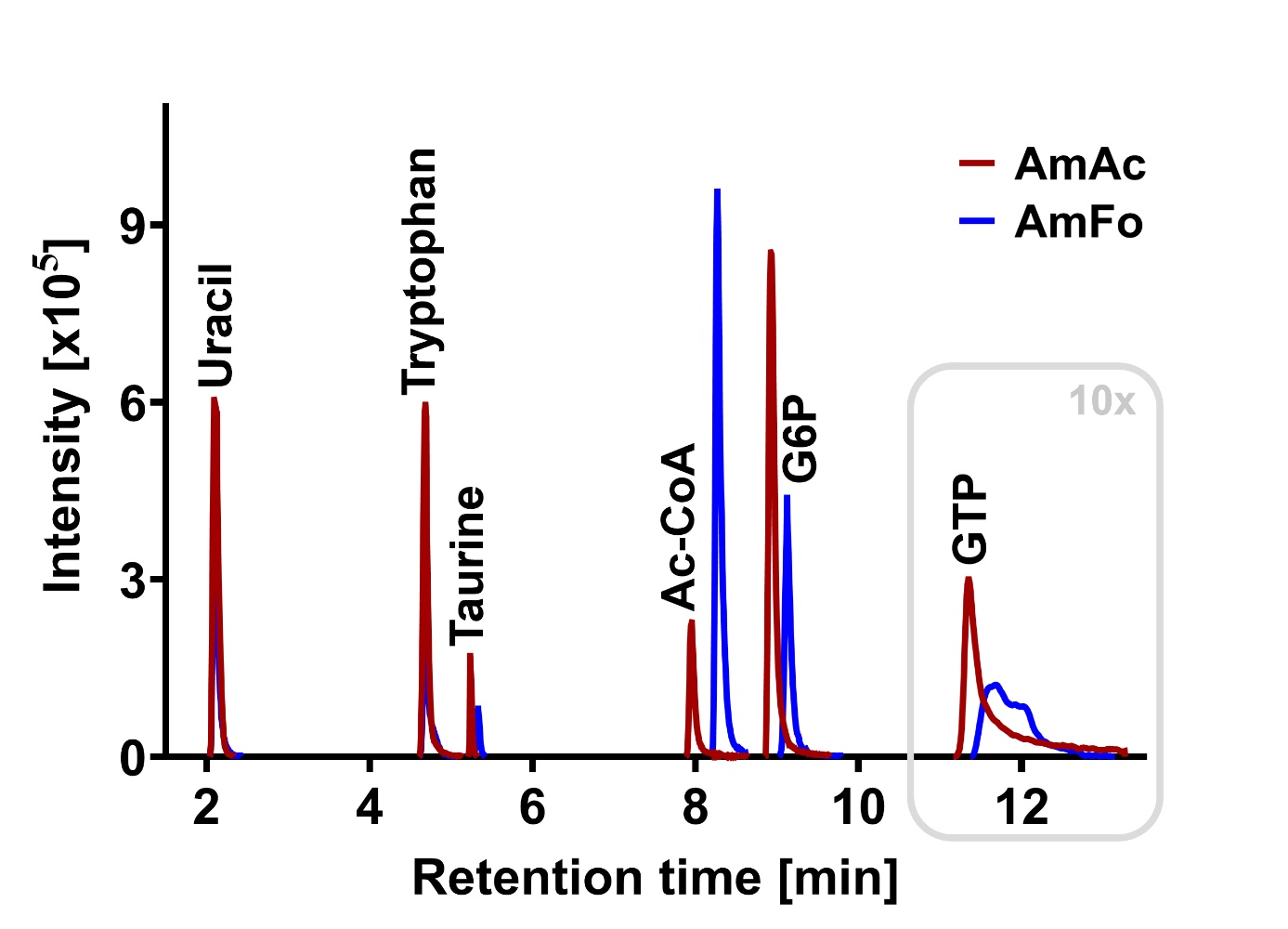


**Figure S4** Effect of pH on the separation of leucine and isoleucine: (**A**) comparison of extracted ion chromatograms of [M-H]^-^ in negative ion mode, (**B**) comparison of extracted ion chromatograms of [M+H]^+^ in positive ion mode

**Figure S5** Effect of pH on the separation of histidine (**A**) comparison of extracted ion chromatograms of [M-H]^-^ in negative ion mode. (**B**) comparison of extracted ion chromatograms of [M+H]^+^ in positive ion mode

**Figure S6** Effect of pH on the separation of tryptophan (**A**) comparison of extracted ion chromatograms of [M-H]^-^ in negative ion mode, (**B**) comparison of extracted ion chromatograms of [M+H]^+^ in positive ion mode

**Figure S7** Effect of pH on the separation of tyrosine (**A**) comparison of extracted ion chromatograms of [M-H]^-^ in negative ion mode, (**B**) comparison of extracted ion chromatograms of [M+H]^+^ in positive ion mode

**Figure S8** Effect of pH on the separation of thymine: (**A**) comparison of extracted ion chromatograms of [M-H]^-^ in negative ion mode, (**B**) comparison of extracted ion chromatograms of [M+H]^+^ in positive ion mode

**Figure S9** Effect of pH on the separation of guanosine: (**A**) comparison of extracted ion chromatograms of [M-H]^-^ in negative ion mode, (**B**) comparison of extracted ion chromatograms of [M+H]^+^ in positive ion mode

**Figure S10** Effect of pH on the separation of 2-deoxyadenosine, comparison of extracted ion chromatograms of [M+H]^+^ in positive ion mode.

**Figure S11** Effect of pH on the separation of glucose-6-phosphate, comparison of extracted ion chromatograms of [M-H]^-^ in negative ion mode

**Figure S12** Effect of pH on the separation of coenzyme A, comparison of extracted ion chromatograms of [M+H]^+^ in positive ion mode

**Figure S13** Effect of pH on the separation of L-carnitine, comparison of extracted ion chromatograms of [M+H]^+^ in positive ion mode

**Figure S14** Effect of pH on the separation of lactose, comparison of extracted ion chromatograms of [M+Na]^+^ in positive ion mode

**Figure S15** Effect of medronic acid concentration (0–7.5 µM) on the signal response of (A) glucose-6-phosphate in negative ion mode and (B) glutamine in positive ion mode


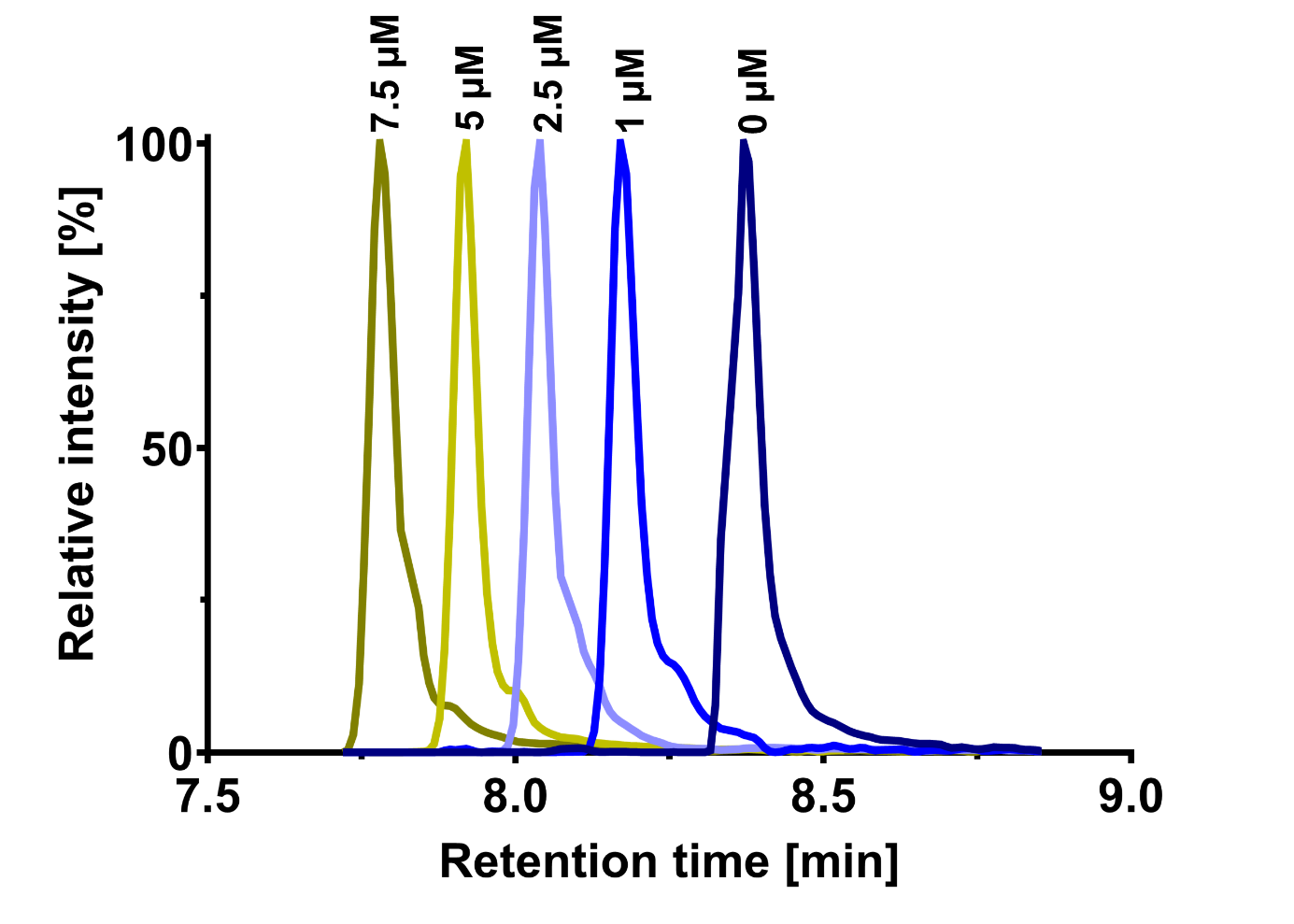


**(A)**


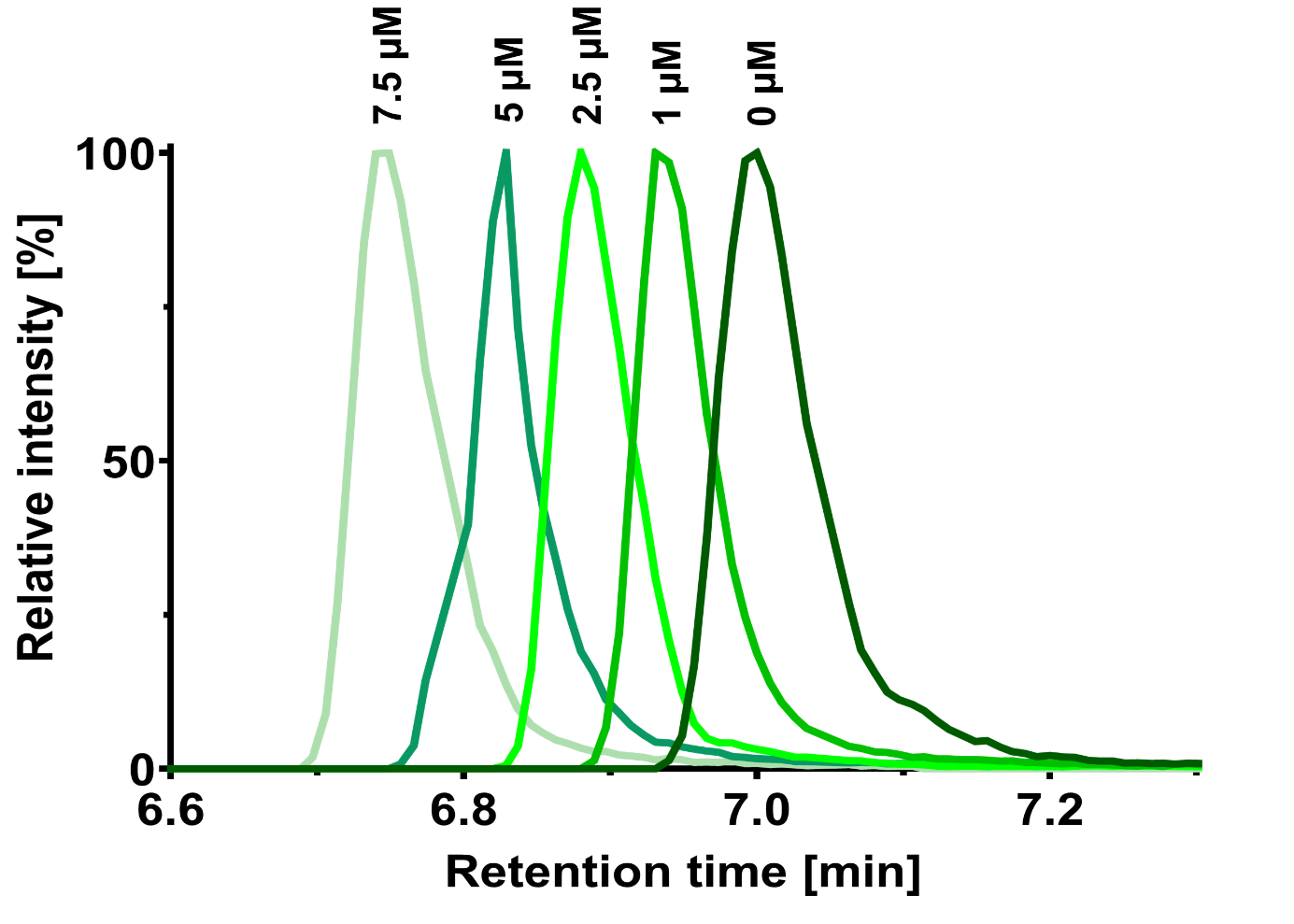


**(B)**

**Figure S16** Characteristic retention behavior for homologous series of acylcarnitines (CAR) and fatty acids (FA), where X represents the carbon number of the saturated acyl chain. The plots show a strong polynomial correlation between retention time and the number of carbons in the acyl chain (X). The acylcarnitine series (A) includes L-carnitine (X=0) and saturated, straight-chain acylcarnitines from acetyl- (X=2) to hexanoyl-carnitine (X=6). The fatty acid series (B) comprises saturated, even-chain fatty acids from X=8 to X=20

**Figure S17** Venn diagram illustrating the distribution and overlap of metabolites annotated in positive and negative ion modes in the analyzed mouse samples


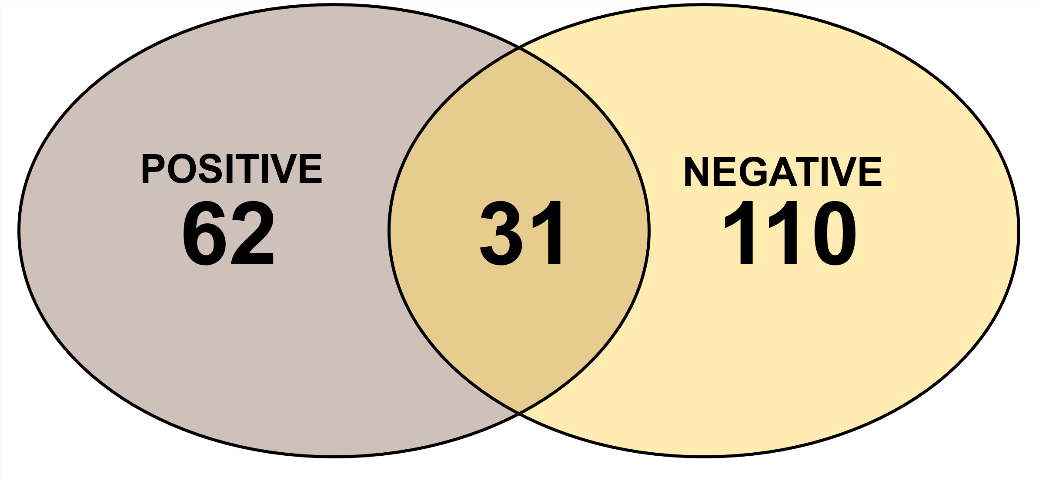

Supplement: Supplementary file 1 — Supplementary Material 1 Supplementary Figures S1-S17, providing comparative chromatograms, boxplots of column performance, data plots from optimization experiments, correlation analysis of homologous series, and a Venn diagram of metabolite distribution. (DOCX 2.14 MB) [file 216_2025_6189_MOESM1_ESM.docx]
